# Enhancement of using combined packing materials on the removal of mixed sulfur compounds in a biotrickling filter and analysis of microbial communities

**DOI:** 10.1186/s12896-019-0540-8

**Published:** 2019-07-25

**Authors:** Xiang Tu, Meiying Xu, Jianjun Li, Enze Li, Rongfang Feng, Gang Zhao, Shaobin Huang, Jun Guo

**Affiliations:** 1 Guangdong Institute of Microbiology, Guangdong Academy of Sciences, Guangzhou, 510070 People’s Republic of China; 2 State Key Laboratory of Applied Microbiology Southern China, Guangzhou, 510070 People’s Republic of China; 3 Guangdong Provincial Key Laboratory of Microbial Culture Collection and Application, Guangzhou, 510070 People’s Republic of China; 4 School of Environment and Energy, South China University of Technology, Guangzhou, 510006 People’s Republic of China

**Keywords:** Biotrickling filter, Packing materials, Malodorous gases, Microbial community, Co-occurrence network

## Abstract

**Background:**

Packing materials is a critical design consideration when employing biological reactor to treat malodorous gases. The acidification of packing bed usually results in a significant drop in the removal efficiency. In the present study, a biotrickling filter (BTF2) packed with plastic balls in the upper layer and with lava rocks in the bottom layer, was proposed to mitigate the acidification.

**Results:**

Results showed that using combined packing materials efficiently enhanced the removal performance of BTF2 when compared with BTF1, which was packed with sole lava rocks. Removal efficiencies of more than 92.5% on four sulfur compounds were achieved in BTF2. Average pH value in its bottom packing bed was about 4.86, significantly higher than that in BTF1 (2.85). Sulfate and elemental sulfur were observed to accumulate more in BTF1 than in BTF2. Analysis of principal coordinate analysis proved that structure of microbial communities in BTF2 changed less after the shutdown but more when the initial pH value was set at 5.5. Network analysis of significant co-occurrence patterns based on the correlations between microbial taxa revealed that BTF2 harbored more diverse microorganisms involving in the bio-oxidation of sulfur compounds and had more complex interactions between microbial species.

**Conclusions:**

Results confirmed that using combined packing materials effectively improved conditions for the growth of microorganisms. The robustness of reactor against acidification, adverse temperature and gas supply shutdown was greatly enhanced. These provided a theoretical basis for using mixed packing materials to improve removal performance.

## Background

Many kinds of malodorous pollutants are generated from various industrial activities [1]. Among them, H_2_S and volatile organic sulfur compounds include methyl mercaptan, ethyl thioether and dimethyl disulfide appear most frequently in malodorous gases and are recognized as the key components due to their extremely low odor threshold [2]. Their release into ambient air cannot be ignored for their threats on health and living quality of nearby residents [3]. Besides, wastewater treatment plant can suffer severely economic loss every year from sulfide-induced corrosion [4].

Biofiltration is commonly considered as a cost-saving and environmentally friendly approach for the treatment of malodorous gases. High removal efficiencies of single malodorous substance were reportedly obtained by biological reactors. However, biological purification of the mixed gases containing multi-component malodorous pollutants remains challenging, predominantly because of the accumulation of sulfate in packing materials arising from the bio-oxidization of sulfur compounds [5]. Severe acidification of packing materials in bioreactor could greatly inhibit the degrading activities of microorganisms, and thus lower the overall removal performance [6].

Strategies available to mitigate the impact of acidification on removal efficiencies of multi-components malodorous gases include washing packing bed [7], inoculating specific degrader [8], using two-stage bioreactor [9] or adding alkaline to neutralize acidity [10]. Here, another method of using combined packing materials was proposed. Previous study found that the bottom bed accounted for major removal of odorous pollutants [11]. However, removal of the upper packing materials from filter bed significantly lowered the overall removal performance of bioreactor. So, it was inferred that the upper packing materials played an important role in buffering the spatial gradient of environmental parameters including pH and sulfate, and then in shaping microbial communities. This has not been elucidated well from the ecological point of view. There are still large uncertainties about interactions among different microbial taxa during the biofiltration process of the mixed malodorous gases.

In the present study, two biotrickling filters (BTFs) using different packing materials were set up. The control was packed with sole lava rocks while the treatment had lava rocks in the bottom layer and plastic balls in the upper layer. The two BTFs were used (1) to assess the effects of combined packing materials on synchronous removal of H_2_S, dimethyl sulfide, ethyl mercaptan and dimethyl disulfide; (2) to prove that combined packing materials help accelerate the leaching of metabolites from the filter bed; (3) to determine the robustness of the BTF employing combined packing materials against adverse environmental conditions; (4) to identify the functional microbial taxa that were responsible for the biotransformation of reduced sulfur compounds; and (5) to decipher the possible mechanisms that underlie the enhancement of combined packing materials on the synchronous removal of mixed sulfur compounds.

## Results

### Removal performances of biotrickling filters for mixed sulfur compounds

Two BTFs were operated in parallel in an effort to compare the effect of using combined packing materials on the removal of mixed sulfur compounds. After the startup, removal efficiencies (REs) of H_2_S rapidly increased near to 100% in both BTFs within four days, and steadily maintained at this level throughout the experiment regardless of varied inlet loading rates. The rapid acclimation BTFs to H_2_S within such short time can be attributed to the inoculums, which originated from a previous reactor treating H_2_S-containing gases.

**Fig. 1:**
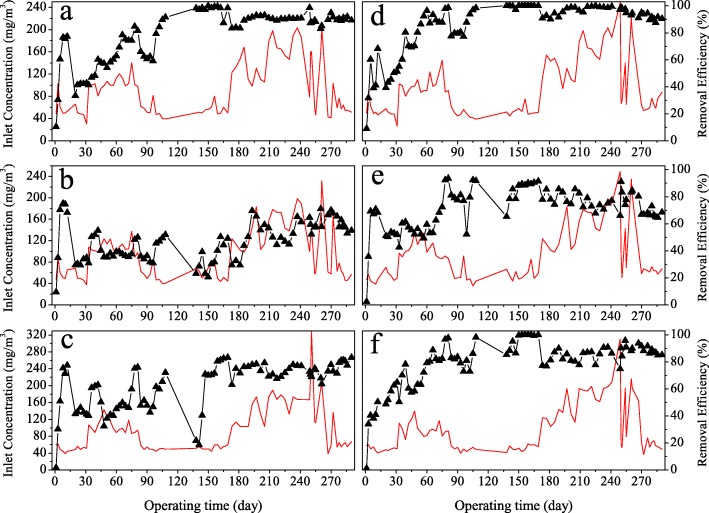
Performance of the biotrickling filters for the removal of volatile organic sulfur compounds: (**a**) EM removal in BTF1, (**b**) DMS removal in BTF1, (**c**) DMDS removal in BTF1, (**d**) EM removal in BTF2, (**e**) DMS removal in BTF2, (**f**) DMDS removal in BTF2

As to volatile organic sulfur compounds (VOSCs), both BTFs took much longer time to adapt. Figure 1 showed the overall removal performances of both BTFs on three VOSCs. Comparatively, BTF2 performed better. REs of ethyl mercaptan, dimethyl sulfide and dimethyl disulfide gradually increased in BTF2. On Day 80, these VOSCs were efficiently removed in BTF2 with 97.8, 92.3 and 96.7% of REs, respectively. BTF1 turned out to be less efficient, and REs of ethyl mercaptan and dimethyl disulfide were 84.5 and 75.1%, respectively. Dimethyl sulfide was among the most difficult to be removed in BTF1. Only 48.2% of dimethyl sulfide was removed on Day 80. Besides, the increase of inlet concentration with up to two times on Day 32 significantly lowered the removal efficiency of dimethyl sulfide in BTF1, which never exceeded 52% throughout the experiment.

There are two possible reasons that both BTFs required longer times to adapt the three organic sulfur compounds. Most likely, acidification of filter bed arising from production of sulfate might lower the activities of some methyl trophic microbes which play crucial roles in the biotransformation of organic sulfur compounds and grow well in neutral environment. Besides, as an important intermediate, the presence of H_2_S could inhibit the bio-oxidation of the three organic sulfur compounds.

### Evaluation of robustness of both BTFs against adverse conditions

Both BTFs were also compared in terms of their robustness against adverse environment conditions including low temperature, shutdown and low pH values of nutrient solution. Temperature is an important factor influencing the removal performance of BTF, particularly in winter. To ensure the successful acclimation, the recycling nutrient solution was heated to about 28 °C from Day 32 to 81. Results showed that higher temperature significantly promoted the removal of all VOSCs in both BTFs. Once the heating was halted from Day 82 to 96, drops of 19.4, 13.8 and 17.1% in REs of ethyl mercaptan, dimethyl sulfide and dimethyl disulfide were observed in BTF2, compared to 20.9, 15.5 and 28.4% seen in BTF1. Apparently, BTF2 was more robust to lower temperature than BTF1 was.

Both BTFs were temporally shut down from Day 109 to 137 due to national holidays. Results showed that about a month idle phase had different effects on the two BTF. No obvious decrease in REs of ethyl mercaptan and H_2_S were observed in both BTFs after shutdown. However, REs of DMS and DMDS decreased drastically in BTF1 from 51.9 and 72.0% to 23.1 and 21.8% respectively. Their restoration to prior REs took about 15 and 30 days, respectively. As to BTF2, only 13% drop in the RE of DMDS was observed, and its full recovery took about one week. The RE of DMS was also affected, decreased from 91.8 to 65.3%. So again, BTF2 was superior to BTF1 against shutdown.

During the last phase of experiment (Day 270–290), pH values of nutrient solution were procedurally reduced from 7.0 to 5.5, resulting in significantly lowered removal performance of BTF2 on all organic sulfur compounds. On the contrary, BTF1 behaved more robust, possibly because it had been acidified already and the community inside got used to low pH.

### Overall removal capacities of both BTFs on four organic sulfur compounds

**Fig. 2:**
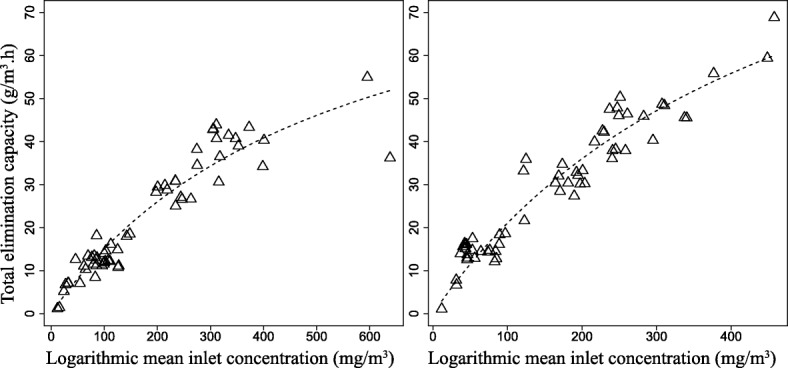
Fitting of Michaelis-Menten equation to the experimental elimination capacities recored in BTF1 (left) and BTF2 (right). Experimental data (triangle) and model data (dash-dot line)

To further assess the overall performances, experimental data from Day 139 to 272 were used to perform the macro kinetics analysis. During this phase, the inlet concentration of individual component changed in a relatively wider range. Figure 2 showed that the Michaelis-Menten type model well fitted to the experimental EC (*P* < 0.001), indicating the two BTFs were not inhibited during this stage. The total EC_max_ calculated from Michaelis-Menten model was 95.0 g/m^3^ h for BTF1 and was 124.3 g/m^3^ h for BTF2. Both estimated total ECs values are very higher than the actual, which was 54.9 for BTF1 and 68.8 g/m^3^.h for BTF2 at inlet loading rates of 74.5 and 77.6 g/m^3^.h, respectively. Macro kinetics showed that neither BTF reached its maximum EC, particularly for H_2_S since the REs of H_2_S in both BTFs were close to 100%. The EC_max_ was the maximum elimination capacity that the BTFs can theoretically achieve in a given range of inlet concentration. However, to accurately predict the behavior of the BTFs in practical application, a pilot-scale experimental should be performed to obtain more empirical data for constructing a reliable kinetic model. Table 1 listed the removal performances of some bioreactors reported in literature. By comparison, the total maximum EC obtained in BTF2 was higher than the BTFs reported by Chen et al. [12–15]. However, the maximum EC varied in a much wide range, depends on the type of packing materials, the composition of waste gases or the configuration of bioreactor.

**Table 1:** Removal performances of some bioreactors reported in literature

Packing materials	Pollutants	Maximum EC (g/m^3^.h)	Removal efficiency (%)	EBRT (s)	Reference
Polyurethane foam	DMDS	86	100	40	[2]
	H_2_S	17	100		
Maifanite	DMDS	19	100	123	[12]
Polyurethane foam	H_2_S	55	80	150	[13]
Polyurethane foam	DMS	23	65	40	[14]
Polyethylene rings	DMS	58	88	60	[15]
	H_2_S	83	100		

### Structure and composition of microbial communities in two BTFs

High-throughput sequencing of bacterial 16S rRNA genes generated an average of 16230 quality sequence reads per sample. All sequence reads were clustered into 1619 operational taxonomic units (OTUs) at 97% similarity, 96.4% of which could be assigned to bacterial or archaeal phylum. The most sequences belonged to bacterial domain, while Archaeal domain only accounted for less than 1.0% of all sequence. Bacterial sequences primarily comprised phylum Proteobacteria (68.7%), followed by phyla Bacteroidetes (15.3%), Actinobacteria (4.9%) and Planctomycetes (1.4%). Among the phylum Proteobacteria, class Beta proteobacteria had highest average relative abundance (30.9%), followed by class Gamma proteobacteria (18.4%) and Alpha proteobacterial (18. 1%). Besides, bacterial Firmicutes and archaeal phylum Euryarcheotic suddenly occurred in BTF1 on Day 150 with very higher relative abundances.

**Fig. 3:**
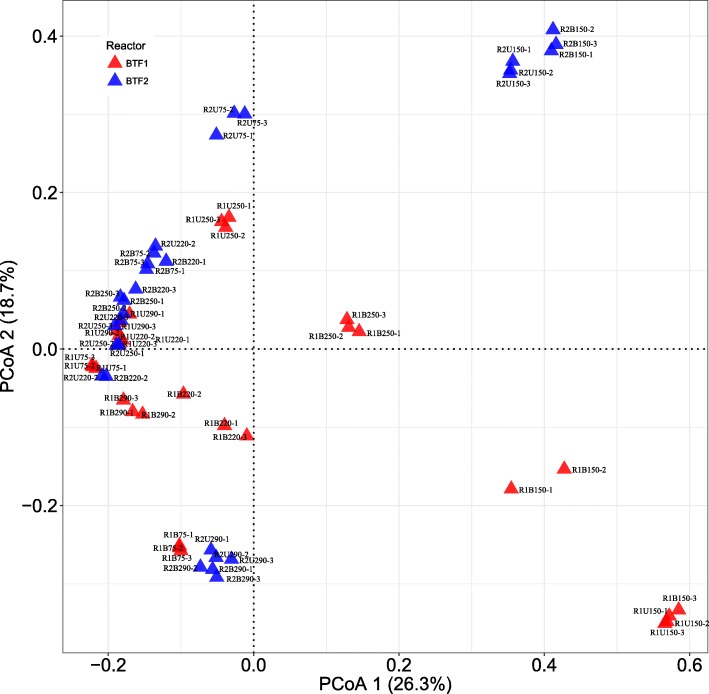
Principal coordinate analysis (PCoA) of microbial communities during different conditions

Principal co-ordinate analysis (PCoA) showed that data could be reduced into two principal components (PC1 and PC2) with combined eigenvalues explaining 45.0% of total variation. Figure 3 demonstrated that microbial communities inside both BTFs dynamically changed with the operating conditions. Samples taken on Day 150 were far distant from others in both BTFs, indicating the structure of microbial communities was seriously affected by the shutdown operation. However, it is evident that using combined packing material introduced a positive effect on the robustness of the microbial community, as seen in BTF2. More than two months later after the shutdown, samples on Day 220 and 250 taken from BTF2 closely grouped with those from Day 75, especially for those from the bottom layer. Comparatively, microbial communities inside BTF1 still sharply diverged even long after BTF1 was restored to the normal operation. Such community changes reconciled with the tendency of removal efficiencies observed in both BTFs. However, BTF2 was much more vulnerable to lower pH conditions than BTF1. Samples collected from BTF2 during acidification period were more distant from those collected on Day 75 and 220 compared to BTF1. It is most likely that microorganisms acclimated within BTF2 favored a neutral condition.

### Correlation network analysis

**Fig. 4:**
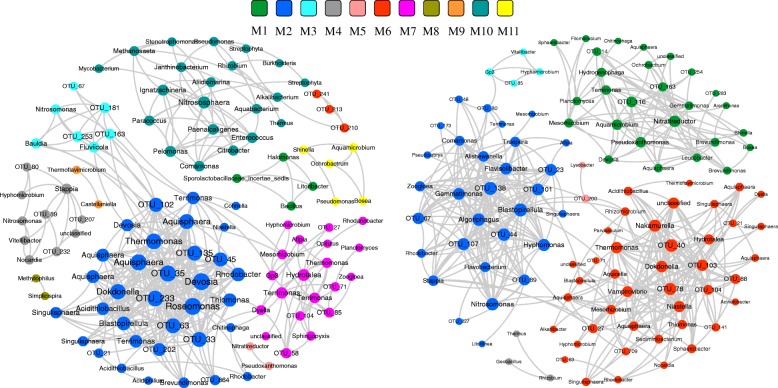
Co-occurrence network showing the correlation between bacterial OTUs in BTF1 (left) and BTF2 (right). A connection stands for a strong (Spearman’s r > 0.8) and significant (Ajusted *p*-value< 0.001) correlation. The size of each node is proportional to the number of connections. Labels according to the taxonomic affiliations. The thickness of each connection between two nodes is proportional to the values of Spearman’s correlation coefficients. Nodes were colored by modularity

Network analysis of taxon co-occurrence patterns can offer new insight into the interconnection of complex microbial communities. Co-occurrence networks were constructed for each BTF based on the Spearman’s correlation (r > 0.8, adjusted *P* values< 0.001). BTF1 network comprised 360 significant associations (edges) of 110 nodes with a clustering coefficient of 0.506 and an overall diameter of 7 (Fig. 4). BTF2 network exhibited 435 strongly positive associations of 108 nodes with an average clustering coefficient of 0.587 and an overall diameter of 9 (Fig. 4).

Both networks could be modularized into several major modules. The modularity of BTF1 and BTF2 network was 0.5590 and 0.6297, respectively. The modularity of more than 0.4 indicates that a network has modular structure [16]. BTF1 network contained 11 modules. Among them, module 2 was the biggest, which consisted of 34 highly interconnected nodes. BTF2 network mainly comprised of six modules. Module 1, 2 and 6 were three major highly dense modules. Although more nodes were included in BTF1 network, BTF2 network had denser connections. So, it was hypothesized that functional groups concerning sulfur oxidation may be enriched in BTF2, which resulted in a highly interconnected network.

## Discussion

Malodorous gases usually contain a variety of pollutants such as hydrogen sulfide, mercaptan and thioether. The complete bio-transformation of these compounds in biofiltration reactor generates sulfate as the main end product [17]. The accumulation of sulfate and the consequent drop in pH values inside packing materials can heavily affect the removal performance. When hydrogen sulfide co-exists with organic sulfur compounds in the odorous gases, some researchers suggested employing two-stage bioprocess with which hydrogen sulfide was removed in the first stage and organic sulfur compounds were in the second stage [10]. However, this would undoubtedly increase the footprint of bioreactor and the consequent construction cost.

Packing materials provide a surface for the biofilm formation. Different packing materials have different physico-chemical properties. Plastic balls and lava rocks are two kind of packing materials commonly used in bioreactor, and greatly differ in many aspects such as roughness, porosity and bulk density. The former features higher porosity and smooth surface by which metabolic product of sulfur compounds could be easily poured out from packing bed. Comparatively, lava rocks have rough surfaces, which retain microbial cells and metabolic products within the micropores.

It has been reported that the bio-degrading activities mainly occurred in the bottom layer of packing bed [11, 18]. As a result, acidification in the bottom layer is more severe, and the growth rates of sulfur-oxidizing microorganisms inhabiting there can be largely reduced. In the present study, lava rocks and plastic balls were used in combination in an effort to improve environmental conditions, and then to create different niches for more diverse microorganisms which could co-operated with each other to degrade the mixed pollutants. Results confirmed that BTF2 packed with combined materials outperformed BTF1 the control, and all target pollutants were efficiently removed in BTF2 with more than 92.5% of REs. Paired t-test indicated a difference (*p* = 0.004, Table 2) in pH values in the bottom layer, with an average 2.85 in BTF1 and 4.86 in BTF2. Mainly owing to the low pH condition, the growth of heterotrophic microbes to some extent may be limited. The pressure drops across the filter bed did not exceed 50 Pa and clogging problem did not occur in both BTFs. Determination of metabolites from Day 251 to 288 also showed that the bottom packing bed of BTF1 accumulated more sulfate than BTF2 (32.3 mg/L versus 21.1 mg/L, *p* < 0.05) (Table 3), and sulfate has been reported to negatively impact the removal performance of biological reactor [18]. Besides, elevated elemental sulfur in BTF1 bottom layer (10.5 versus 2.1 mg/cm^3^-packing) suggested an incomplete bio-oxidation of sulfur compounds in BTF1. It is most likely that low pH reduced the solubility of oxygen and pollutants as reported by Charnnok et al. [19].

**Table 2:** pH values tested inside different filter layers of the two BTFs

Sampling sites	BTF	Operating time (day)							*p*-value
		12	42	75	108	164	251	273	
Upper	BTF1	6.74	6.68	7.05	7.34	7.51	6.53	6.99	0.5581
	BTF2	6.71	7.66	7.03	7.43	6.90	6.99	6.93	
Middle	BTF1	4.13	4.27	4.67	4.89	6.80	6.05	6.60	0.3443
	BTF2	5.14	7.07	6.01	6.51	6.05	5.86	4.94	
Bottom	BTF1	1.92	2.33	2.58	2.61	3.55	3.48	3.47	0.0004
	BTF2	4.07	5.5	4.14	5.01	5.80	5.22	4.26

**Table 3:** Sulfate concentration tested inside different filter layers of the two BTFs (mg/L)

location	day	251	264	274	288	*p*-value
Upper	BTF1	12.1	16.9	19.3	39.8	0.021
	BTF2	9.5	11.5	14.9	31.6	
Middle	BTF1	13.3	12.8	17.4	25.4	0.018
	BTF2	8.4	10.8	10.6	20.5	
Bottom	BTF1	18.7	21.0	27.0	62.5	0.031
	BTF2	10.5	14.8	15.9	43.0

**Table 4:** Experimental schedule and operating conditions

Phase	Time	Average inlet concentration (mg/m^3^)				Temperature (°C)	pH
	day	H_2_S	EM	DMS	DMDS		
1	1–31	101.0 ± 10.8	58.2 ± 17.2	59.3 ± 17.2	57.4 ± 15.5	19.1 ± 4.1	7.0
2	32–81	188.4 ± 56.1	102.5 ± 14.5	106.1 ± 13.9	100.9 ± 16.7	28.0 ± 0	7.0
3	82–96	109.7 ± 21.4	56.3 ± 9.6	62.0 ± 15.5	51.0 ± 5.4	8.0 ± 4.9	7.0
4	97–108	98.7 ± 1.4	44.0 ± 5.0	43.5 ± 4.1	48.8 ± 3.1	28.0 ± 0	7.0
5	109–138*	–	–	–	–	–	–
6	139–272	174.2 ± 93.1	96.3 ± 74.5	99.1 ± 78.5	105.5 ± 83.6	26.9 ± 1.3	7.0
7	273–290	118.1 ± 16.5	59.1 ± 13.2	60.7 ± 11.4	59.8 ± 10.9	26.9 ± 1.3	5.5 ± 0.8

*Both BTFs were shut down during this phase

Sulfate and elemental sulfur were the main end products from sulfur compounds biodegradation since no other intermediates were detected in both BTFs. Given that the average concentrations of sulfate in both BTFs were noticeably higher than that of elemental sulfur, it is likely that most sulfur compounds were bio-converted into sulfate. The occurrence of local anoxic zone within filter bed was the leading cause of the slight accumulation of elemental sulfur. Sulfur compounds could be incompletely bio-oxidized to elemental sulfur when oxygen supply is limited. Theoretical Gibbs free energy changes of DMS, EM and DMDS bio-oxidation were calculated according to stoichiometric Eqs. 1, 2, 3. Results indicate that the bio-oxidation of the three organic sulfur compounds is thermodynamically favorable. BTF heavily relies on microorganisms to degrade various gaseous pollutants. In this study, all reduced sulfur compounds can serve as carbon or energy source to support the growth of microorganisms. Most energy generated in these processes is captured by microorganisms in the form of ATP for their anabolisms.

1 $$ {\mathrm{CH}}_3-\mathrm{S}-{\mathrm{CH}}_3+{5\mathrm{O}}_2\to {2\mathrm{CO}}_2+{2\mathrm{H}}_2\mathrm{O}+{\mathrm{H}}_2{\mathrm{SO}}_4\ {\Delta \mathrm{G}}^0=-1948.9\ \mathrm{KJ}/\mathrm{mol} $$

2 $$ {\mathrm{CH}}_3-{\mathrm{CH}}_2-\mathrm{SH}+{5\mathrm{O}}_2\to {2\mathrm{CO}}_2+{2\mathrm{H}}_2\mathrm{O}+{\mathrm{H}}_2{\mathrm{SO}}_4\ {\Delta \mathrm{G}}^0=-2021.0\ \mathrm{KJ}/\mathrm{mol} $$

3 $$ {\mathrm{CH}}_3-\mathrm{S}-\mathrm{S}-{\mathrm{CH}}_3+13/2\ {\mathrm{O}}_2\to {2\mathrm{CO}}_2+{\mathrm{H}}_2\mathrm{O}+{2\mathrm{H}}_2{\mathrm{SO}}_4\ {\Delta \mathrm{G}}^0=-2391.3\ \mathrm{KJ}/\mathrm{mol} $$

Using oxygen as an electron acceptor, these compounds could be biodegraded to sulfate by phylogenetically diverse microorganisms following an oxidative pathway. For instance, DMS can be oxidized by methyltransferase or DMS monooxygenases to methanethiol. Methanethiol is subsequently degraded by methanethiol oxidase to produce sulfide. Finally, Sulfide is converted to sulfate via sulfite by the well-known SOX system [20].

The biodegradation pathway of EM and DMDS has not been well deciphered in literature. A few reports found that the EM degradation was initiated by formation of diethyl disulfide [21, 22]. Although the main end products were confirmed as elemental sulfur and sulfate based on the calculation of sulfur mass balance, the subsequent oxidation route of diethyl disulfide remains unclear, and enzymes and genes involved were not reported.

Together with these results confirmed that using combined packing materials successfully prevented severe acidification in the packing materials. Consequently, both BTFs differed in the structure of microbial diversity. Apart from samples taken on Day 150, the shannon index was significantly higher in BTF2 than in BTF1 (3.13 vs 2.69, *p* < 0.01). Microorganisms able to degrade sulfur compounds are extremely diverse, including autotrophs such as *Thiobacillus* and *Acidithiobacillus*, and heterotrophs microorganisms such as *Bacillus*, *Xanthobacter* and *Hyphomicrobium* [14, 15, 23, 24]. Most of them prefer neutral conditions. For instance, the drop pH value could lower the specific growth rate of genus *Hyphomicrobium* [25]. Only members of genus *Acidithiobacillus* could oxidize hydrogen sulfide in extremely acidic conditions [15].

OUT_6, OUT_7 and OUT_8 were the three key OUTs in BTF1 communities with relative abundance of 1.78, 2.86 and 2.48% on average. OUT_6 was assigned to genus *Thiomonas*, while OUT_7 and 8 were assigned to genus *Acidithiobacillus*. Both genera are (i) well-known sulfur-oxidizers, (ii) capable of chemo lithotrophic growth on various sulfur compounds [26, 27], and (iii) often reported to occur inside bioreactors treating sulfur-containing waste gases [17, 28]. Co-occurrence network analysis demonstrated that the three co-occurred in Module 2 of BTF1 network. So, it can be inferred that this module had potential links with the removal of sulfur compounds in BTF1. It is worth noting that the most abundant OUT (OUT_1) with relative abundance of 26.29% on average, were not constructed into BTF1 networks, possibly because it is autotrophs whose survival rarely relies on microbial incorporations. OUT_1 was assigned to genus *Thiobacillus*, and most of members of *Thiobacillus* are obligate autotrophs using elemental sulfur and reduced inorganic sulfur compounds as energy source [29]. The consistent predominance of *Thiobacillus* might account for the effective removal of H_2_S in both BTFs. H_2_S removal prior to other organic sulfur compounds is critical since that H_2_S is the main intermediate metabolite of organic sulfur compounds and has an inhibitive effect on the oxidation of the latter [30, 31]. OUT_2 and OUT_4 were other two predominant OTUs of not being constructed into BTF1 network. Their average relative abundances in BTF1 reached to 8.89 and 3.66%, respectively. OUT_2 belonged to the genus *Halothiobacillus*, while OUT_4 was classified as member of genus *Hyphomicrobium*. Members of both genera were reportedly the important sulfur-oxidizing microbes [32, 33].

Sulfur-degraders inside BTF2 network were mainly clustered in Module 6, which contained 42 highly interconnected nodes. This module resembles Module 2 of BTF1 network in ecological function but is much denser, suggesting that BTF2 harbored more diverse microorganisms involving in the removal of sulfur compounds. OTU_6 (*Thiomonas*) and OTU_8 (*Acidithiobacillus*) also predominated in BTF2 with average relative abundance of 2.52 and 0.69%, respectively. In addition, OTU_15 (*Hyphomicrobium*) co-occurred with OTU_6 and OTU_8 in Module 6, which was another bacterial taxon that dominated inside BTF2 (4.03%). Methylotrophic *Hyphomicrobium* is considered as an important degrader of organic sulfur compounds [34], so its co-occurrence with other sulfur-oxidizing bacteria might contribute a lot to the simultaneous removal of H_2_S and organic sulfur compounds. OTU_1 (*Thiobacillus*) was also the most predominant microbial taxon (28.9%) but failed to be constructed in BTF2 network. Unlike BTF1, other top abundant taxa in BTF2 were successfully included in the network, indicating that more type of microorganisms took actively part in the degradation of mixed sulfur compounds in BTF2, resulting in a much-enhanced cooperative network.

Together, this study proved that using combined packing materials efficiently enhanced the synchronous removal of mixed sulfur compounds by facilitating the elimination of acidic product out from filter bed. This strategy offers a promising alternative for the control of malodorous gases. Nonetheless, there is still a pressing need to further understand the ecological mechanisms that underlie the synergistic removal of complex sulfur compounds in one-stage bioreactor. To achieve the goal, functional genes that are responsible for degradation of each sulfur compound need to be quantitatively analyzed and should be linked to specific microbial species. Besides, the understanding of the removal profile of each malodorous pollutant and the spatial distribution of microbial community along the filter bed will aid in identifying the roles played by the upper and bottom packing materials in removing complex pollutants.

Malodorous gas that generated from a real emission source is usually complicated. The composition and the concentration of malodorous gases often change. To better use this strategy, pilot-scale experiments are required to be conducted in some industrial emission source. For industrial application, the volume ratio of the upper/bottom packing materials should be adjusted correspondently, depending on the composition and the concentration of malodorous gas. Other materials having similar physico-chemical properties also can be used as packing materials of biotrickling filters in the same way.

## Conclusions

The removal of malodorous pollutants mostly occurred in the bottom packing materials of biotrickling filter. However, the roles of upper packing materials in buffering the distribution of pH values along the packing bed are usually neglected. Results confirmed that using different packing materials in combination greatly enhanced the removal of mixed sulfur compounds. More favorable environmental condition for the growth of microorganisms was created in BTF2, resulting in a denser microbial correlation network in which diverse microorganisms took part in the degradation of mixed sulfur compounds. The robustness of microbial community against environmental stress such as shutdown and low temperature, was also enhanced in BTF2. These findings shed some light on the biofiltration of complex malodorous gases.

## Methods

### Biotrickling filters setup and operation

**Fig. 5:**
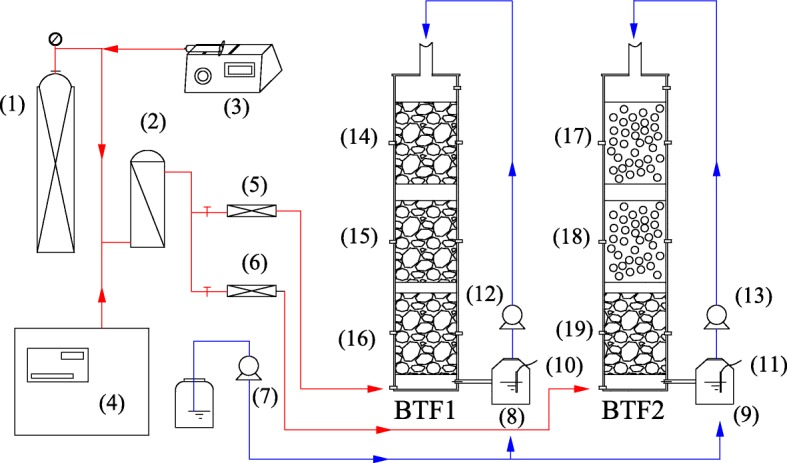
Schematic diagram of the biotrickling filters (1) H_2_S cylinder, (2) mixing chamber, (3) syringe pump, (4) air compressor, (5 and 6) gas flowmeter, (7) NaOH dosing pump, (8 and 9) nutrient tank, (10 and 11) pH probe, (12 and 13) peristaltic pump, (14–19) filter material sampling ports

**Fig. 6:**
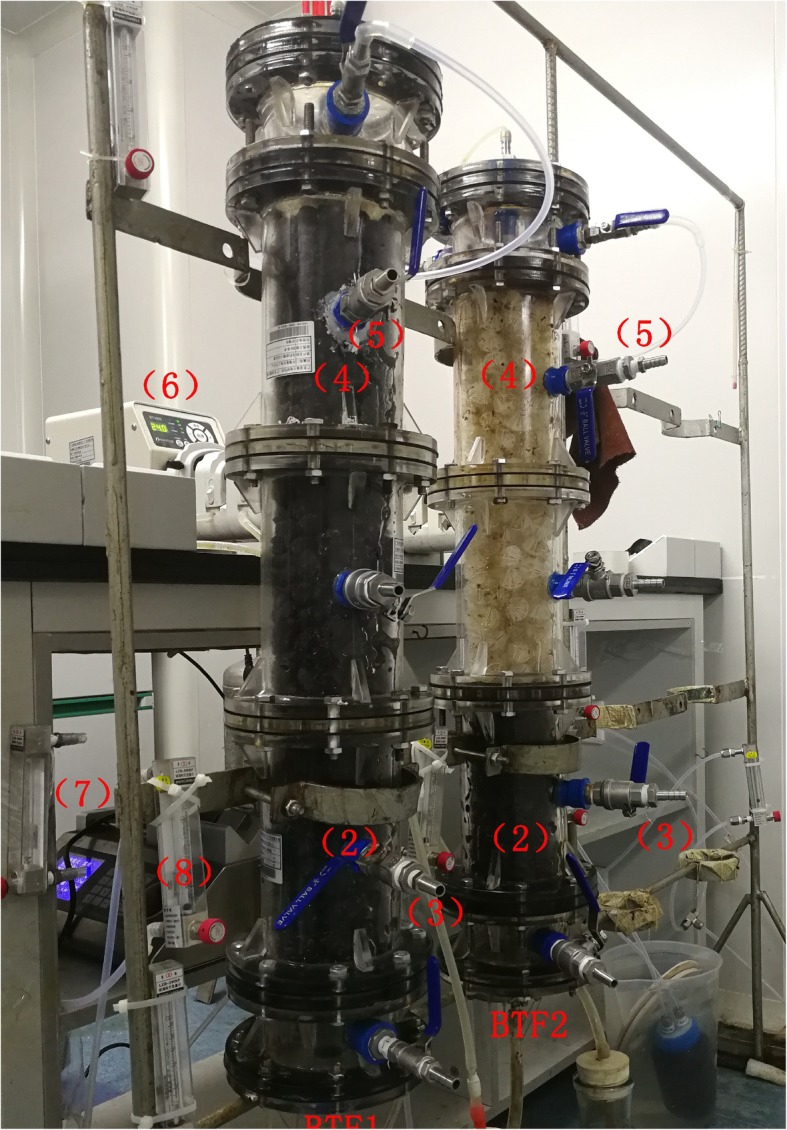
Actual photograph of the biotrickling filters (1) nutrient solution, (2) bottom filter bed, (3 and 5) filter materials sampling ports, (4) upper filter bed, (6) peristaltic pump, (7) syringe pump, (8) gas flowmeter

Two identical laboratory-scale biotrickling filters (BTFs) were employed, named BTF1 and BTF2, respectively. The schematic diagram and the actual photograph of the BTFs was showed in Figs. 5 and 6, respectively. Each was composed of three columns in the stacked configuration, with an inner diameter of 10 cm and a total height of 38 cm. The resulted packing volume of each BTF was about 3 L. BTF1 was packed with lava rocks, which have particle sizes of 6–10 mm and bulk density of 0.57 g/cm^3^. BTF2 was packed with lava rocks in the bottom layer, while with plastic ball in the upper layer. Plastic balls are polyhedral and hollow spheres which are made of polypropylene and are 2.5 cm in diameter. The bulk density, porosity, and specific surface area of plastic ball are 80 kg/m^3^, 92% and 460 m^2^/m^3^, respectively. For generating the malodorous gases, compressed air was split into a major and a minor airstream. Mixed liquid of three organic sulfur compounds including dimethyl sulfide (DMS), ethyl mercaptan (EM) and dimethyl disulfide (DMDS), was injected into the minor air stream via a syringe pump (ShenChen, SPLab02, China). H_2_S vapors were also directly introduced from a gas cylinder into the minor air stream. Two air streams were finally mixed in a chamber and were then fed to both BTFs in the up-flow mode.

Both BTFs were operated at an empty bed residence time (EBRT) of 60 s across the experiment. The experimental schedule and operating conditions were listed in Table 4. Initial inoculum originated from a previous biotrickling filter treating H_2_S-containing gases [35]. Nutrient solution was stored in a holding-tank, and evenly sprayed into packing column by using a peristaltic pump (ShenChen, BT300, China). Nutrient solution contained NH_4_Cl 2.5 g, Na_2_HPO_4_ 1.0 g, KH_2_PO_4_ 0.7 g, MgSO_4_ 0.05 g, CaCl_2_ 0.015 g per liter in water. The pH values of nutrient solution were adjusted at 7.0 by automatically adding alkaline by using a dosing pump (SKEO, AKS600, Italy). Nutrient solution was periodically renewed to ensure sufficient nutrient and moisture for the growth of microorganisms.

### Gas-phase determination

Gas-phase concentrations were measured with a gas chromatograph (Shimadzu GC-2010, Japan) equipped with an FPD detector. The column used was a GS-Gas Pro capillary column (30 m × 0.32 mm × 1.0 μm, Agilent Technologies, USA). Gas samples were taken using Tedlar bags of 2 L. Total volume of 100 μL was injected into the GC using a gastight syringe. The injector and detector temperature were set at 70 °C and 250 °C, respectively. The GC oven temperature was programmed as follows: initial temperature of 80 °C for 2 min, increase to 250 °C at 10 °C min^− 1^ and maintain for 5 min.

Macro-kinetic model was used to evaluate the elimination capacities (ECs) of each BTF. The ECs commonly follow a behavior that can be adequately described by a Michaelis-Menten model type [36]. This model is constructed based on ECs and is relatively simple and useful in predicting the removal performance of practical engineering.

$$ \mathrm{EC}={\mathrm{EC}}_{\mathrm{max}}\frac{\mathrm{Cln}}{\mathrm{Ks}+\mathrm{Cln}} $$

Where EC_max_ (g.m^3^/h) is the maximum elimination capacity, Cln (g/m^3^) is the logarithmic average of the inlet and outlet concentrations of pollutants in the gas phase and Ks (g/m^3^) is the saturation constant.

### DNA extraction and high-throughput sequencing

2 g of packing materials were sterilely collected in triplicate from the upper and bottom filter layer of each BTF on Day 75, 150, 220, 250 and 290. Packing materials were mixed with 10 mL phosphate buffer (NaCl 8 g/L, KCl 0.2 g/L, Na_2_HPO_4_ 1.42 g/L, KH_2_PO_4_ 0.27 g/L). After gently shaking, biofilms were completely detached from the surface by ultrasonication for 10 min. The suspensions were collected and centrifuged for 10 min at 10,000 g, and the resulted pellet was collected for the DNA extraction.

Genomic DNA was extracted using the MO-BIO PowerSoil® DNA Isolation Kit (MO BIO Laboratories, Inc., USA) according to the manufacturer’s instructions. Universal primers 515F (5′-GTG CCA GCM GCCGCG GTA A-3′) and 909R (5′- CCC CGY CAA TTC MTT TRA GT − 3′) with 12 nt unique barcodes were used to amplify the V4 and V5 hypervariable regions of the 16S rRNA gene for pyrosequencing using a MiSeq sequencer.

### Data analysis

The raw data were firstly quality-filtered with QIIME Pipeline to remove reads that did not meet the desired quality [37]. All sequence reads were trimmed and assigned to the corresponding samples based on their barcodes. Multiple steps were taken to trim the sequences, such as the removal of sequences < 200 bp, and average base quality score Q < 25. Any chimeric sequences were identified and removed using Uchime algorithm. Sequences were clustered into operational taxonomic units (OTUs) using a 97% similarity. Taxonomic assignment was performed using the RDP classifier at a confidence level of 80%. All samples were randomly rarefied to an equal sequence number to neutralize the bias of varied sequencing depth.

Principal coordinate analysis (PCoA) was performed using vegan and ggplot2 packages in R software. PCoA provides information regarding the largest source of variation in the data and allows the observation of similarities and differences between samples.

Co-occurrence networks were inferred for each BTF based on the Spearman correlation matrix between bacterial OTUs. Valid connection indicates a strong (r > 0.8) and significant (*p* < 0.001) Spearman’s correlation. To minimize pairwise comparisons and reduce the network complexity, only OTUs with more than 0.05% of relative abundance were considered. Both the correlation matrix and significance matrix were calculated using Hmisc package in R. To reduce the chances of obtaining false-positive results, *P*-values were adjusted by FDR using the BH method with the multtest package in R [38]. The network was visualized using Cytoscape 3.5.1 [39]. Modular structure of highly interconnected nodes was detected with multi-level algorithm using igraph package in R. A number of parameters (e.g. number of nodes and edges, betweenness centrality, clustering and modularity coefficient) were calculated using igraph too. OTUs with maximum betweenness centrality score were considered as keystone species [40].

## Data Availability

All data generated or analyzed during this study are included in this published article.

## Abbreviations

BTF: Biotrickling filter
DMDS: Dimethyl disulfide
DMS: Dimethyl sulfide
EBRT: Empty bed residence time
EC: Elimination capacity
EM: Ethyl mercaptan
OTUs: Operational taxonomic units
PCoA: Principal co-ordinate analysis
RE: Removal efficiency
